# Does Oral Health-Related Quality of Life Differ by Income Group? Findings from a Nationally Representative Survey

**DOI:** 10.3390/ijerph191710826

**Published:** 2022-08-30

**Authors:** André Hajek, Hans-Helmut König, Benedikt Kretzler, Larissa Zwar, Berit Lieske, Udo Seedorf, Carolin Walther, Ghazal Aarabi

**Affiliations:** 1 Department of Health Economics and Health Services Research, University Medical Center Hamburg-Eppendorf, Hamburg Center for Health Economics, 20246 Hamburg, Germany; 2 Department of Periodontics, Preventive and Restorative Dentistry, University Medical Center Hamburg-Eppendorf, 20251 Hamburg, Germany

**Keywords:** oral health, oral health-related quality of life, income, low income, high income, income poverty, mouth diseases

## Abstract

Objectives: Clarify the association between income group and oral health-related quality of life. Methods: Data were used from a nationally representative online survey with *n* = 3075 individuals. It was conducted in late Summer 2021. The established Oral Health Impact Profile (OHIP-G5) was used to measure oral health-related quality of life. The income group (household net income) was used as key independent variable. It was adjusted for several covariates. Full-information maximum likelihood was used to address missing values. Results: Individuals in the lowest income decile had a lower oral health-related quality of life (Cohen’s d = −0.34) compared to individuals in the second to ninth income deciles. Individuals in the highest income decile had a higher oral health-related quality of life (Cohen’s d = 0.20) compared to individuals in the second to ninth income deciles. Consequently, there was a medium difference (Cohen’s d = 0.53) between individuals in the lowest income decile and individuals in the highest income decile. Additionally, multiple linear regressions showed significant differences between individuals in the lowest income decile and individuals in the second to ninth income deciles (β = 0.72, *p* < 0.01). In contrast, only marginal significant differences were identified between individuals in the second to ninth income deciles and individuals in the highest income decile (β = −0.28, *p* < 0.10). Conclusions: The current study particularly stressed the association between low income and low oral health-related quality of life in the general adult population. Increasing oral health-related quality of life in individuals with low income is a major issue which should be targeted.

## 1. Introduction

Oral health may be defined as “the health of the teeth, gums, and the oral-facial system that allows us to smile, speak, and chew” [1]. Some of the most common diseases that impair oral health are caries, gingivitis, periodontal disease, and oral cancer. More than a quarter (26%) of adults in the United States has untreated caries [1]. Severe periodontitis affects about 9% of adults and nearly half (46%) of all adults aged 30 or more years have mild or moderate forms of periodontal disease [2]. Although tooth loss among adults aged 65–74 years follows declining trends over time, large disparities exist among some population groups [3]. Untreated caries and periodontitis lead to tooth loss and periodontitis is associated with other chronic diseases such as diabetes, heart disease, and stroke, which share risk factors such as tobacco smoking and high intake of sugar-rich foods and beverages [4].

Low individual/household income is associated with oral conditions, including severe periodontitis [5,6,7], dental caries prevalence and any caries experience [8], tooth loss [9], and oral cancer [10]. However, less is known regarding the potential association between income and Oral Health-Related Quality of Life (OHRQoL). Individual/household income is a key aspect of the socio-economic status (SES), which is already proven to be strongly associated with the OHRQoL [11,12]. Focusing on key elements of this complex construct deepens our knowledge in different health outcomes across populations.

Previous results show that low parental income is associated with worse OHRQoL among children [13]. OHRQoL is an important concept in dentistry because it is a multidimensional construct that provides a subjective rating of an individual’s oral health, functional well-being, emotional well-being, expectations and satisfaction with care, and sense of self [14].

To what extent psychosocial and behavioral characteristics contribute to associations between low individual or household income and oral health is largely unknown at present. Since OHRQoL is an integral part of general health and well-being, this study aims to clarify the potential association between income group and OHRQoL based on a nationally representative study from Germany. Well-designed epidemiological studies can address knowledge gaps concerning income-related oral health inequalities.

## 2. Materials and Methods

### 2.1. Sample

We used data from a nationally representative survey. In total, *n* = 3075 individuals aged 18 to 70 years residing in Germany were included. A power calculation was performed prior to the study. The data were collected from late August to early September 2021. The market research company respondi recruited the participants by means of their own online panel. Recruitment was conducted in a way ensuring representativeness (quota-based) regarding age group, gender and federal state. In total, 14,000 individuals were contacted. Since this was an online sample, a potential sample selection bias could not be calculated.

All participants included in this study provided informed consent. Approval for the study was provided by the Local Psychological Ethics Committee of the Center for Psychosocial Medicine of the University Medical Center Hamburg-Eppendorf (number: LPEK-0356). Our study is in accordance with the ethical standards laid down in the 1964 Declaration of Helsinki and its later amendments.

### 2.2. Outcome: Oral Health-Related Quality of Life

The well-established Oral Health Impact Profile (OHIP-G5) [15], which consists of five items, was used to quantify OHRQoL. It covers four dimensions: (1) oral function, (2) orofacial pain, (3) appearance and (4) psychosocial impact. The sum score ranges from 0 to 20. It is worth emphasizing that *higher* scores correspond to *lower* OHRQoL. In our study, Cronbach’s alpha was 0.85. Good to very good psychometric properties have also been shown elsewhere [15].

### 2.3. Independent Variables

The key independent variable was the monthly household net income group: under EUR 500, 500 EUR to lower than EUR 1000, EUR 1000 to lower than EUR 1500, EUR 1500 to lower than EUR 2000, EUR 2000 to lower than EUR 2500, EUR 2500 to lower than EUR 3000, EUR 3000 to lower than EUR 3500, EUR 3500 to lower than EUR 4000, EUR 4000 to lower than EUR 4500, EUR 4500 to lower than EUR 5000, EUR 5000 to lower than EUR 6000, EUR 6000 to lower than EUR 8000, EUR 8000 or more.

Income was also trichotomized (1: *lowest income decile* including a monthly household net income lower than EUR 1000; 2: *second to ninth income deciles* including a monthly household net income from EUR 1000 up to lower than EUR 4500; 3: *highest income decile* including a monthly household net income of EUR 4500 and above).

The following covariates were included: gender, age, marital status (married, living together with spouse; married, not living together with spouse; single; widowed; divorced), educational level (upper secondary school; qualification for applied upper secondary school; polytechnic secondary school; intermediate secondary school; lower secondary school; currently in school training/education; without school-leaving qualification), chronic diseases (absence of chronic diseases; presence of one or more chronic diseases) and self-rated health (from 1 = very bad to 5 = very good).

### 2.4. Statistical Analysis

Sample characteristics are first shown (stratified by income group). Thereafter, the effect sizes (Cohen’s d) were given (income group and OHRQoL). Subsequently, multiple linear regressions were conducted to examine the association between income group and OHRQoL. Since 314 individuals (10.2%) did not report their income, (1) linear regressions were computed with listwise deletion to address missing values *and*–our preferred choice–(2) linear regressions were computed with full information maximum likelihood (FIML) [16] to address the issue of missing data.

The level of significance was set at *p* < 0.05. Stata 16.1 (Stata Corp., College Station, TX, USA) was used for performing statistical analyses.

## 3. Results

### 3.1. Sample Characteristics Stratified by Income Group and Effect Sizes

The average age in the total sample was 44.5 years (with SD: 14.8 years, 18 to 70 years). In total, 51.1% were female.

Individuals in the lowest income decile had an average OHRQoL score of 3.4 (SD: 4.1), whereas individuals in the second to ninth income deciles had an average OHRQoL score of 2.2 (SD: 3.3) and individuals in the highest income decile had an average OHRQoL score of 1.6 (3.0). The differences regarding OHRQoL between income groups were significant (*p* < 0.001). Please see Table 1 and Table 2 for further details.

With regard to effect sizes, individuals in the lowest income decile had lower OHRQoL (Cohen’s d = −0.34) compared to individuals in the second to ninth income deciles. Individuals in the highest income decile had a higher OHRQoL (Cohen’s d = 0.20) compared to individuals in the second to ninth income deciles. Consequently, there was a medium difference (Cohen’s d = 0.53) in terms of OHRQoL between individuals in the lowest income decile and individuals in the highest income decile.

### 3.2. Regression Analysis

Findings of multiple linear regressions are shown in Table 3 (second column: with listwise deletion to address missing values; third column: with FIML to address missing values). For example, with FIML to address missing values and after adjusting for several covariates, linear regressions showed higher OHIP-G5 scores (worth repeating: corresponding to a lower OHRQoL) among individuals in the lowest income decile compared to individuals in the second to ninth income deciles (β = 0.72, *p* < 0.01). However, only marginal significant differences were identified between individuals in the second to ninth income deciles and individuals in the highest income decile (β = −0.28, *p* < 0.10).

In Supplementary Table S1, the covariates are also displayed. In Supplementary Tables S2–S6, findings of multiple linear regressions with the different OHIP-G5 components as outcomes are shown.

## 4. Discussion

### 4.1. Main Findings

Based on a large representative study, our goal was to clarify the association between income group and OHRQoL. Our study shows that particularly individuals with low income reported low OHRQoL. These findings were also supported by regression analysis, including adjustments for gender, age, marital status, education, employment status, presence of chronic diseases, and self-rated health.

### 4.2. Previous Research and Possible Explanations

In total, our observations agree with the few previous results from other cross-sectional studies. In a recently published systematic review on the association of SES and OHRQoL, the meta-analysis of 75 studies showed that people with lower SES had significantly worse OHRQoL [11]. For example, findings from a cross-sectional study in Hong Kong demonstrated lower household income, divided into income groups, was significantly associated with higher score in the OHIP-14, therefore representing worse OHRQoL [17]. Similarly, results from the 4th National Oral Health Survey among over 4000 Chinese adults showed that participants with higher household income per capita had better OHRQoL [18]. The negative impact of low household income on a person’s OHRQoL has also been reported among adolescents [12,19]. Data from the Adult Dental Health Survey (ADHS; conducted in the United Kingdom) 2009 showed that OHRQoL was positively associated with education [20,21,22], income [21,22], and social class [20,22]. Earlier studies on dentate adults from the ADHS 1998 showed social gradients by income and social class, but not education, in adjusted models [23,24]. Moreover, results from the Office of National Statistics (ONS) surveys, conducted in the United Kingdom, demonstrated that non-manual labor social class participants had better OHRQoL than those belonging to manual labor social classes [25,26,27]. Data on older adults (aged 50+ years) from the English Longitudinal Study of Ageing demonstrated inequalities in OHRQoL by education, income, and wealth [28,29,30].

Income-related oral health inequalities become even more visible when controlling the different components of OHIP G5. Lower household income is associated with worse oral function [31], the disadvantages members of society with the lowest income present the highest prevalence of orofacial pain [32], and they experience discrimination due to unideal dental appearance [33].

Cross-sectional studies and meta-analyses demonstrated that low individual/household income was related to the prevalence of several oral conditions known to lower OHRQoL, including severe periodontitis [5,6,7,34], dental caries prevalence and any caries experience [8], traumatic dental injuries [35], and oral cancer [35]. Thus, it may be assumed that the individuals’ objective oral health status contributes to OHRQoL because low income is a predictor of poor oral health. However, OHRQoL is a multidimensional construct that also includes subjective ratings of functional well-being, emotional well-being, expectations and satisfaction with care, and sense of self [14]. To what extent psychosocial and behavioral characteristics contribute to the observed association between low individual income and OHRQoL should be a topic for future research.

### 4.3. Strengths and Limitations

Data were used from a large sample which corresponds to the distribution of age bracket, federal state and gender in the German adult population. To address missing values, a FIML approach was used. OHRQoL was operationalized using the established OHIP-G5. It should be acknowledged that the online survey could only be performed in the German language, which could exclude individuals with poor skills in German language. This could lead to an underestimation of the true association. Additionally, an online bias is possible which means that our findings refer solely to individuals with internet access. Individuals without internet access may reflect individuals with a very low income, which could affect our results. Moreover, only the income group, but not the actual income (continuously measured) was used. This was done to reduce the number of missing values. The household size was not included due to reasons of data availability. Moreover, our present study has a cross-sectional design that makes it difficult to establish the directionality of the association.

## 5. Conclusions

The current study particularly stressed the association between low income and low OHRQoL in the general adult population. Increasing OHRQoL in individuals with low income is a major issue which should be targeted.

## Figures and Tables

**Table 1:** Sample characteristics stratified by income group.

Variables	Lowest Income Decile	Second to Ninth Income Deciles	Highest Income Decile	*p*-Value
	N = 328	N = 2006	N = 427	
Gender				0.15
Men	166 (50.6%)	987 (49.2%)	236 (55.3%)	
Women	161 (49.1%)	1017 (50.7%)	191 (44.7%)	
Diverse	1 (0.3%)	2 (0.1%)	0 (0.0%)	
Age	41.3 (16.8)	45.4 (14.5)	44.6 (13.0)	<0.001
Marital status				<0.001
Single/Divorced/Widowed/Married, not living together with spouse	272 (82.9%)	837 (41.7%)	58 (13.6%)	
Married, living together with spouse	56 (17.1%)	1169 (58.3%)	369 (86.4%)	
Highest educational degree				<0.001
Upper secondary school	114 (34.8%)	772 (38.5%)	282 (66.0%)	
Qualification for applied upper secondary school	37 (11.3%)	210 (10.5%)	44 (10.3%)	
Polytechnic Secondary School	26 (7.9%)	123 (6.1%)	7 (1.6%)	
Intermediate Secondary School	80 (24.4%)	648 (32.3%)	82 (19.2%)	
Lower Secondary School	65 (19.8%)	243 (12.1%)	11 (2.6%)	
Currently in school training/education	2 (0.6%)	6 (0.3%)	0 (0.0%)	
Without school-leaving qualification	4 (1.2%)	4 (0.2%)	1 (0.2%)	
Employment status				<0.001
Full-time employed	23 (7.0%)	1012 (50.4%)	307 (71.9%)	
Retired	81 (24.7%)	355 (17.7%)	30 (7.0%)	
Other	224 (68.3%)	639 (31.9%)	90 (21.1%)	
Chronic diseases				<0.001
Absence of chronic diseases	160 (48.8%)	1106 (55.1%)	288 (67.4%)	
Presence of at least one chronic disease	168 (51.2%)	900 (44.9%)	139 (32.6%)	
Self-rated health (1 = very bad to 5 = very good)	3.3 (1.0)	3.6 (0.9)	3.9 (0.8)	<0.001
Oral health-related quality of life (OHIP-G5; from 0 to 20, with higher scores indicating lower oral health-related quality of life)	3.4 (4.1)	2.2 (3.3)	1.6 (3.0)	<0.001

Notes: Oneway anova or Chi^2^-tests were conducted, as appropriate.

**Table 2:** Average oral health-related quality of life (stratified by household net income group).

Household Net Income Group	Oral Health-Related Quality of Life (Mean and SD in Parentheses)
under EUR 500 (*n* = 104)	3.1 (3.9)
500 EUR to lower than EUR 1000 (*n* = 224)	3.5 (4.1)
EUR 1000 to lower than EUR 1500 (*n* = 291)	2.8 (3.6)
EUR 1500 to lower than EUR 2000 (*n* = 361)	2.4 (3.5)
EUR 2000 to lower than EUR 2500 (*n* = 364)	2.3 (3.4)
EUR 2500 to lower than EUR 3000 (*n* = 330)	2.2 (3.2)
EUR 3000 to lower than EUR 3500 (*n* = 272)	1.9 (3.2)
EUR 3500 to lower than EUR 4000 (*n* = 247)	1.8 (3.1)
EUR 4000 to lower than EUR 4500 (*n* = 141)	2.1 (3.2)
EUR 4500 to lower than EUR 5000 (*n* = 180)	1.7 (2.8)
EUR 5000 to lower than EUR 6000 (*n* = 131)	1.2 (2.8)
EUR 6000 to lower than EUR 8000 (*n* = 85)	1.5 (3.1)
EUR 8000 or more (*n* = 31)	2.1 (4.3)
Not reported (*n* = 314)	1.7 (2.5)

Notes: According to a oneway anova, group differences were significant (*p* < 0.001).

**Table 3:** Determinants of oral health-related quality of life. Results of multiple linear regressions.

Independent Variables	Oral Health-Related Quality of Life–with Listwise Deletion to Address Missing Values	Oral Health-Related Quality of Life–with FIML to Address Missing Values
Lowest income decile (Ref.: Second to ninth income deciles)	0.75 ** (0.25)	0.72 ** (0.24)
Highest income decile	−0.29 + (0.17)	−0.28 + (0.16)
Covariates	√	√
Observations	2761	3075
R^2^	0.07	0.06

Notes: Unstandardized beta-coefficients are displayed; robust standard errors (SE) in parentheses; ** *p* < 0.01, + *p* < 0.10; Covariates include gender, age, marital status, education, employment status, presence of chronic diseases and self-rated health. Thus, a tick symbol (√) was used.

## Data Availability

The datasets generated and/or analysed during the current study are not publicly available due to legal restrictions but are available from the corresponding author on reasonable request.

## Supplementary Materials

The following supporting information can be downloaded at: https://www.mdpi.com/article/10.3390/ijerph191710826/s1, Table S1. Determinants of oral health-related quality of life (all covariates are displayed). Results of multiple linear regressions. Table S2. Determinants of ‘Oral function: Difficulty chewing foods’. Results of multiple linear regressions. Table S3. Determinants of ‘Oral function: Less flavor in food’. Results of multiple linear regressions. Table S4. Determinants of ‘Appearance: Unfomfortable about appearance’. Results of multiple linear regressions. Table S5. Determinants of ‘Oral function: Difficulty chewing foods’. Results of multiple linear regressions. Table S6. Determinants of ‘Psychosocial impact: Difficulty doing your usual jobs’. Results of multiple linear regressions.
