# Supplementary material for: Does Oral Health-Related Quality of Life Differ by Income Group? Findings from a Nationally Representative Survey
Source: Int J Environ Res Public Health. 2022 Aug 30;19(17):10826. doi: 10.3390/ijerph191710826 (PMC9518370; doi:10.3390/ijerph191710826)
Supplement: Supplementary file 1 [file ijerph-19-10826-s001.zip › ijerph-1852192-supplementary.pdf]

International Journal of
Environmental Research and Public Health

MDPI

Supplementary Table S1. Determinants of oral health-related quality of life (all covariates are displayed). Results of multiple linear regressions.

Independent Variables	Oral Health-Related Quality of Life–with Listwise Deletion to Address Missing Values	Oral Health-Related Quality of Life–with FIML to Address Missing Values
Lowest income decile (Ref.: Second to ninth income decile)	0.75 **	0.72 **
	(0.25)	(0.24)
Highest income decile	−0.29 +	−0.28 +
	(0.17)	(0.16)
Gender: -Women (Ref.: Men)	−0.26 +	−0.23 +
	(0.14)	(0.13)
- Diverse	0.16	0.25
	(1.86)	(1.83)
Age	−0.02 ***	−0.02 ***
	(0.01)	(0.01)
Marital status: -Married, living together with spouse (Ref.: Single/Divorced/Widowed/Married, not living together with spouse)	−0.11	−0.09
	(0.14)	(0.13)
Highest educational degree: -Qualification for applied upper secondary school (Ref.: upper secondary school)	0.15	0.20
	(0.21)	(0.19)
- Polytechnic Secondary School	0.43	0.43
	(0.32)	(0.30)
- Intermediate Secondary School	0.31 *	0.25 +
	(0.15)	(0.14)
- Lower Secondary School	0.54 *	0.48 *
	(0.24)	(0.23)
- Currently in school training/education	1.99	1.70
	(1.40)	(1.28)
- Without school-leaving qualification	2.44	2.51
	(1.91)	(1.90)
Employment status: -Retired (Ref.: Full-time employed)	0.07	0.06
	(0.21)	(0.20)
- Other	−0.10	−0.17
	(0.16)	(0.14)
Chronic diseases: Presence of at least one chronic disease (Absence of chronic diseases)	0.45 **	0.40 **
	(0.15)	(0.14)
Self-rated health (1 = very bad to 5 = very good)	−0.66 ***	−0.68 ***
	(0.10)	(0.09)
Constant	5.50 ***	5.53 ***
	(0.49)	(0.45)
Observations	2761	3075
R^2	0.07	0.06

Unstandardized beta-coefficients are displayed; robust standard errors (SE) in parentheses; *** $p < 0.001$, ** $p < 0.01$, * $p < 0.05$, + $p < 0.10$; Covariates include gender, age, marital status, education, employment status, presence of chronic diseases and self-rated health.

Supplementary Table S2. Determinants of 'Oral function: Difficulty chewing foods'. Results of multiple linear regressions.

**Independent Variables**	**Oral Function: Difficulty Chewing Foods–with Listwise Deletion to Address Missing Values**	**Oral Function: Difficulty Chewing Foods–with FIML to Address Missing Values**
Lowest income decile (Ref.: Second to ninth income decile)	0.18 ** (0.07)	0.17 ** (0.06)
Highest income decile	−0.02 (0.05)	−0.02 (0.05)
Covariates	√	√
Observations	2761	3075
R^2	0.05	0.05

Unstandardized beta-coefficients are displayed; robust standard errors (SE) in parentheses; ** $p < 0.01$; Covariates include gender, age, marital status, education, employment status, presence of chronic diseases and self-rated health. Thus, a tick symbol (√) was used.

Supplementary Table S3. Determinants of 'Oral function: Less flavor in food'. Results of multiple linear regressions.

**Independent variables**	**Oral Function: Less Flavor in Food–with Listwise Deletion to Address Missing Values**	**Oral Function: Less flavor in Food–with FIML to Address Missing Values**
Lowest income decile (Ref.: Second to ninth income decile)	0.08 (0.05)	0.07 (0.05)
Highest income decile	−0.06 (0.04)	−0.06 (0.04)
Covariates	√	√
Observations	2761	3075
R^2	0.03	0.03

Unstandardized beta-coefficients are displayed; robust standard errors (SE) in parentheses; Covariates include gender, age, marital status, education, employment status, presence of chronic diseases and self-rated health. Thus, a tick symbol (√) was used.

Supplementary Table S4. Determinants of 'Appearance: Unfomfortable about appearance'. Results of multiple linear regressions.

**Independent Variables**	**Appearance: Unfomfortable about Appearance–with Listwise Deletion to Address Missing Values**	**Appearance: Unfomfortable about Appearance–with FIML to Address Missing Values**
Lowest income decile (Ref.: Second to ninth income decile)	0.18 ** (0.07)	0.18 ** (0.06)
Highest income decile	−0.05 (0.04)	−0.05 (0.04)
Covariates	√	√
Observations	2761	3075
R^2	0.05	0.05

Unstandardized beta-coefficients are displayed; robust standard errors (SE) in parentheses; ** $p < 0.01$; Covariates include gender, age, marital status, education, employment status, presence of chronic diseases and self-rated health. Thus, a tick symbol (√) was used.

Supplementary Table S5. Determinants of 'Oral function: Difficulty chewing foods'. Results of multiple linear regressions.

**Independent Variables**	**Oral Function: Difficulty Chewing Foods–with Listwise Deletion to Address Missing Values**	**Oral Function: Difficulty Chewing Foods–with FIML to Address Missing Values**
Lowest income decile (Ref.: Second to ninth income decile)	0.17 * (0.08)	0.17 * (0.08)
Highest income decile	−0.10 * (0.05)	−0.10 * (0.05)
Covariates	√	√
Observations	2761	3075
R^2	0.06	0.06

Unstandardized beta-coefficients are displayed; robust standard errors (SE) in parentheses; * $p < 0.05$; Covariates include gender, age, marital status, education, employment status, presence of chronic diseases and self-rated health. Thus, a tick symbol (√) was used.

Supplementary Table S6. Determinants of 'Psychosocial impact: Difficulty doing your usual jobs'. Results of multiple linear regressions.

Independent Variables	Psychosocial Impact: Difficulty Doing Your Usual Jobs–with Listwise Deletion to Address Missing Values	Psychosocial Impact: Difficulty Doing Your Usual Jobs–with FIML to Address Missing Values
Lowest income decile (Ref.: Second to ninth income decile)	0.15 ** (0.05)	0.14 ** (0.05)
Highest income decile	−0.06 + (0.03)	−0.05 + (0.03)
Covariates	√	√
Observations	2761	3075
R^2	0.04	0.04

Unstandardized beta-coefficients are displayed; robust standard errors (SE) in parentheses; ** $p < 0.01$, + $p < 0.10$; Covariates include gender, age, marital status, education, employment status, presence of chronic diseases and self-rated health. Thus, a tick symbol (√) was used.
